# Use and Perceptions of a Campus Food Pantry Among Food Insecure College Students An Exploratory Study from Appalachia

**DOI:** 10.13023/jah.0202.02

**Published:** 2020-04-15

**Authors:** Laura H. McArthur, Kimberly S. Fasczewski, Alisha R. Farris, Miranda Petrone

**Affiliations:** Department of Nutrition and Health Care Management, Appalachian State University; Department of Health and Exercise Science, Appalachian State University; Department of Nutrition and Health Care Management, Appalachian State University; Department of Nutrition and Health Care Management, Appalachian State University

**Keywords:** Appalachia, college students, food insecurity, campus food pantries

## Abstract

**Introduction:**

Food insecurity has emerged as a public health problem among college students in Appalachia, jeopardizing their physical, mental, and emotional health and academic success. Campus food pantries have been established in this region, but no data are available concerning student use or perception of services.

**Purpose:**

This study measured use and perceptions of a campus food pantry by students at a mid-sized university in rural North Carolina.

**Methods:**

An online questionnaire collected behavioral and perceptual data, and follow-up interviews explored these variables. Descriptive statistics with significance at p<0.05 and thematic analytical procedures were used.

**Results:**

Questionnaires were submitted by 896 of 6000 recruited students (14.9%), and four students granted interviews. Food insecurity affected 437 (48.8%) of participants, of whom 76 (17.4%) were pantry shoppers. Shoppers (n = 94) were 27.7% males, 65.1% females, and 7.2% non-cisgender, 63.8% non-Hispanic white, 84.5% undergraduate, and 14.3% graduate students. Reasons for non-pantry use by food insecure students included: others need it more (30.1%) and feel embarrassed (20.7%). Benefits of pantry use were: spent more on necessities (56.4%) and job performance improved (18.1%). Shoppers perceived the pantry’s physical environment most favorably and food offerings less favorably.

**Implications:**

The low use of the campus food pantry by food insecure students suggests that these students may be jeopardizing their physical and mental health and academic success. Greater efforts by faculty, academic advisers, and student leaders are needed to promote pantry use and decrease the associated stigma.

## INTRODUCTION

Food security is defined as having consistent access, in socially acceptable ways, to safe and nutritious food that promotes an active and healthy life.^1^ The Adult Food Security Survey Module (AFSSM), developed by the United States Department of Agriculture Economic Research Service (USDAERS), is a validated tool administered annually to measure the food security status of the adult population. Items are worded to distinguish between four levels of food security as follows: high (no food-access problems or limitations), marginal (one or two indications, typically of anxiety over food sufficiency or shortage of food, with little or no changes in diets or food intake), low (reduced diet quality, variety, or desirability, with little or no indication of reduced food intake), and very low (disrupted eating patterns and reduced food intake). Persons whose scores fall in the high or marginal categories are classified as food secure and those whose scores fall in the low or very low categories are classified as food insecure.^1^

Prevalence data from the USDAERS indicate that approximately 37.2 million adults were low food secure at some time during 2018, including 9.5 million who were very low food secure.^2^ Groups in 2018 with food insecurity rates above the national average of 11.1% were households with children, single parent households, men and women living alone, black, non-Hispanics, Hispanics, residents of the southern and southeastern regions, households with incomes below 185% of the poverty threshold, persons living in food deserts, and persons with physical disabilities and mental health disorders.^2^–^6^ The reported outcomes of prolonged food insecurity for children and adults include physical and mental health problems (e.g., obesity, type 2 diabetes, heart disease, and depression),^7^,^8^ impaired cognitive functionality,^9^,^10^ and poor growth and development in children and adolescents.^11^

Ample evidence from 2-year and 4-year public and private post-secondary institutions nationwide indicates that food insecurity is widespread among college students,^12^ with rates ranging from 14.8% at an urban university in Alabama^1^ to 59.0% at a rural university in Oregon.^14^ High rates of food insecurity were also found among students attending seven colleges and universities in the Appalachian region, with rates ranging from 22.4% to 51.8%.^15^ The sociodemographic and behavioral factors most commonly reported for food insecure college students are: older age, receiving food assistance, having less money to buy food, identifying with a minority race/ethnic group, being employed while in school, on-campus residence, having lower self-efficacy for cooking cost-effective, nutritious meals, and having less time to prepare food.^13^–^17^ In addition to the unfavorable health impacts of food insecurity identified for the general population, college students experiencing food deprivation also show suboptimal academic performance, as reflected in a low grade point average (GPA), and higher rates of school drop-out and an increased risk for depression, anxiety, and stress compared to their food secure peers.^17^–^19^

Campus food pantries are widespread on college and university campuses in response to the student food insecurity problem. As of January 2020, 650 post-secondary institutions were members of the College and University Food Bank Alliance, including schools in Appalachia.^20^ Yet, few studies were located that report student use of these facilities, and, to our knowledge, no studies have been published that assess student use of food pantries on campuses in Appalachia. Therefore, findings from two studies conducted in other regions of the country are summarized here. El Zein et al.^21^ found that only 38% of food insecure students attending the University of Florida used their campus food pantry. Barriers for pantry use were social stigma, insufficient information about pantry policies, self-identity, and inconvenient pantry hours. Rouse reported that 69% of the food insecure students at the University of California, Santa Barbara used the campus food bank, and that greater than 75% of pantry shoppers were highly satisfied with the nutritious quality and with the quantity of available foods and customer service, but were less satisfied with the variety of food offerings, food sanitation, and pantry size (see Additional Files).

The present study was conducted at Appalachian State University (AppState), a mid-sized university located in the western region of North Carolina, an area with high rates of poverty, obesity, and food insecurity rates ranging from 13% to 16.8%.^2^,^22^,^23^ During the spring 2016 semester the rate of student food insecurity at AppState was estimated at 46.2%,^24^ and in the fall of that year the Free Store and Food Pantry was established by the Office of Sustainability. The food pantry section of the facility is available to students, staff, and faculty and is operated much like a grocery store to offer shoppers a familiar retail experience. Food offerings include various grain and cereal products (including fresh bread), canned, refrigerated, and frozen foods, and seasonal, locally-grown fruits and vegetables. Inventory is maintained through cash donations and donations from local bakeries, farmer’s markets, and grocery stores. Recipe cards for low-cost healthy meals and snacks are also available, and pantry staff offer information about applying for federal and state food assistance programs. The pantry is conveniently located in the basement of a residence hall and is opened Monday through Friday from 8:00 a.m. to 5:00 p.m. After two years, data were needed to evaluate use and perceptions of pantry services to improve future operations. To assist with that task, and to contribute to the literature about student use of campus food pantries in Appalachia, the aims of this mixed-methods, cross-sectional study were to measure pantry use and perceptions (i.e., benefits of pantry use, physical environment, food offerings, customer service, and feelings when shopping at the pantry) among undergraduate and graduate students enrolled at the university during the spring, 2018 semester.

## METHODS

### Participant Recruitment

A computer-generated randomized sample of 6000 first-year through graduate students received electronic recruitment letters for participation in an on-line survey, followed by an email reminder one and two weeks later.^25^ Students interested in learning more about the study clicked on a link in the recruitment letter that took them to a screen containing an informed consent letter, and those who wished to proceed clicked an "accept" button that took them to the questionnaire. No compensation was offered for participation. This research was approved by the Institutional Review Board at the university.

### Questionnaire

Quantitative data were collected using an anonymous online questionnaire administered through Qualtrics survey software (Qualtrics, November 22, 2015, Provo UT: 2015). Food security status was measured using the Adult Food Security Survey Module (AFSSM).1. Next, two yes/no questions asked whether the students were aware of the campus food pantry, and whether they ever shopped there, respectively. The students who had never patronized the pantry selected, from a list of 16 reasons, those that explained why they had never accessed food from that facility. These reasons were categorized as: personal (4 reasons), food offerings (4 reasons), food access (5 reasons), and awareness (3 reasons). All reasons are listed in Table 1. Pantry shoppers estimated how frequently they had shopped since enrolling at the university by selecting either only once, once or twice/semester, once or twice/month, once or twice/week, or more than twice/week. Perceived benefits of pantry use were identified from the following list: got information about food assistance; able to spend more money on other necessities like rent, or utilities; class attendance improved, grades improved; able to stay enrolled; and improved job performance. The shoppers next completed a pantry attributes rating scale with four response options ranging from strongly disagree to strongly agree. These attributes were categorized as: physical environment (4 attributes), food offerings (6 attributes), and customer service (3 attributes).

The final questions addressed sociodemographic, academic, and cooking variables. Progress in school was measured with a four-item Academic Progress Scale (APS) where students self-rated on the following variables: (1) overall progress in school (including graduating on time), (2) class attendance, (3) attention span in class, and (4) understanding of concepts taught. Response options were poor, fair, good, or excellent. These same responses assessed perceived cooking skills. Frequency of cooking for self or others was assessed with the responses never, seldom, sometimes, or often, and a yes/no item assessed access to basic cooking equipment. All listings and scales were developed by the authors with guidance from the food security literature^12^–^19^, and each provided an “other” option.

Content validity was determined by two nutrition professors. The questionnaire was pilot tested online with a computer-generated randomized sample of 50 undergraduate and graduate students who did not participate in the final study. Student feedback indicated that the links and buttons operated accurately and that the screens displayed an appropriate amount of text. Input also prompted deletion of items that were never selected.

### Interviews

Requests for 15-minute face-to-face interviews appeared as a final yes/no item on the questionnaire. The purpose of these interviews was to add supplemental understanding to the survey data to help clarify the experiences of the students. Interviews were semi-structured and were conducted in the privacy of the PI’s office by the PI and by a graduate student in the Nutrition Department. Only students who had shopped at the pantry at least once were interviewed. Note-taking rather than audio recording was used to capture responses because this method was thought to put the students at greater ease. Verbatim responses were taken down, transcripts were made available to the students for verification, and all data were deidentified. No compensation was offered for interviews. Questions addressed: (1) benefits from pantry use, (2) perceptions of pantry attributes, (3) feelings when shopping, and (4) suggestions for improvements.

### Data Analyses

Quantitative data were analyzed using SPSS (SPSS Statistics for Windows, Version 24, Armonk, NY: IBM Corp. 2016). AFSSM data were scored such that 0 (zero) affirmative responses reflected high, 1–2 marginal, 3–5 low, and 6–10 very low food security, and students who scored from zero to 2 were classified as food secure while those who scored from 3 to 10 were classified as food insecure.^1^ Frequency counts and percentages were calculated on all data. The APS and the item assessing perceived cooking skills were scored by allotting 1 point to the poor, 2 to the fair, 3 to the good, and 4 to the excellent responses. The item concerning frequency of cooking for self or others was scored by allotting 1 point to the never, 2 to the seldom, 3 to the sometimes, and 4 points to the often responses. Chi-square analyses compared proportions of pantry users on sociodemographic, academic, and cooking variables, and correlational analyses examined associations between the students’ AFSSM scores and their GPAs and APS scores. Data were not analyzed based on race/ethnicity due to the small amount of diversity in the sample, reflecting the enrollment at the university. Statistical significance was p≤0.05.

The handwritten interview notes were individually read by two separate researchers and thematic content was assigned independently. The themes were driven by the responses and informed by the research questions. Researchers then met and agree on the themes.^26^ Responses were then assigned to one of the four themes addressed in the interview based on how closely the content of the students’ comments matched the concepts addressed in the theme, and the number of pertinent responses under each theme category were counted. All categorization was checked for consensus by two researchers for agreement before meaning was determined. Due to the logistics of the research, the interview data were used only as supplemental information and were not intended to do anything more than add context to the survey data and inform the interpretation of the results. More detailed qualitative analysis with in-depth interviews would be needed for a true understanding of the personal experiences of students using the food pantry; this was beyond the scope of the current study.

## RESULTS

### Participant Profile

Questionnaires were submitted by 896 students (14.9%) of the 6000 who were recruited, and three male pantry shoppers (M1, M2, and M3) and one female shopper (F1) granted interviews. Table 2 shows the findings regarding sociodemographic, academic, and cooking variables for all participants and for the group of pantry shoppers. Individual responses were included if participants answered the pantry use data, even if some demographic information was omitted. Consequently, reported percentages are based on the number of respondents for each question and vary accordingly.

In summary, about 70% of the entire sample was female, their mean age was 21.1 years (±3.4, range 18 to 59), and approximately 80% were non-Hispanic whites. AFSSM scores indicated that approximately 50% of the students were food insecure. Additionally, about 55% were employed, 25% participated in an on-campus meal plan, and 1% in a state or federal food assistance program. The most frequently selected yearly family income and personal monthly income brackets, respectively, were $50,000 to $74,999 and $0.0 to $500. About 90% of the students were enrolled full-time, 80% were undergraduates, and 25% lived on-campus.

The students’ mean GPA was 3.41 points out of a possible 4.0 points, and there was a significant negative correlation between AFSSM scores and GPA (r = −0.205, p=0<0.01). The students’ mean APS score was 12.9 points (±2.35) out of a possible 16 points, and a significant negative correlation was also found between their AFSSM and APS scores (r = −0.379, p<0.01). Findings concerning cooking variables indicated that about 40% of the students rated their cooking skills as good or excellent, 40% cooked for themselves or others sometimes or often, and 60% had access to basic cooking equipment.

### Profile of Pantry Shoppers

The majority of the students, 581 (64.8%) were aware of the pantry, and 94 students (10.5%) had shopped at the pantry at least once. The shoppers were about two-thirds female, their mean age was 21.8 years (± 4.3, range 18 to 59), and two-thirds were non-Hispanic whites. Additionally, about 50% were employed, 20% participated in an on-campus meal plan, and 4% participated in a state or federal food assistance program. The most frequently selected yearly family income and personal monthly income brackets, respectively, were $50,000 to $74,999 and $0.00 to $500. About 85% (80) of the 94 pantry shoppers were full-time students, 85% (80) were undergraduates, and 20% (19) lived on-campus. Findings concerning cooking variables revealed that about 45% of the shoppers rated their cooking skills as good or excellent, 45% cooked for themselves or others sometimes or often, and 55% had access to basic cooking equipment. Only 18 of the pantry shoppers were classified as food secure and among the cohort of 437 food insecure students, 76 (17.4%) had ever shopped at the pantry.

### Use of the Campus Food Pantry

Table 1 shows the frequency counts and percentages for the reasons why the food insecure students never frequented the pantry. The three reasons selected most often were: others need it more than I do, selected by about one-third of the food insecure students; I feel embarrassed asking for help accessing food, selected by about 20%; and I don’t know how to ask for help accessing food, selected by about 10%. The frequency of pantry shopping among the 76 food insecure students was: 4.8% only once, 5.2% once or twice/semester, 3.1% once or twice/month, and 1.7% once or twice/week. Two students commented on how they felt while shopping at the pantry “I felt weird being in there because I didn't need it as much as others do. There is some negative stigma to using the pantry. I would like to see the University work on getting rid of stigma because no one is alone in the struggle." (M2) and the other commented “I felt kind of sorry for students who had to go and get food; even that they had to walk through the doors.” (F1)

### Perceptions of the Campus Food Pantry

Perceptions of the campus food pantry were explored using 13 items (see Table 3 for a complete list) divided into three sub-categories (physical environment, food offerings, customer service). The two perceived benefits of pantry shopping most often selected were: able to spend more money on other necessities like rent, utilities (n = 53, 56.4%) and improved job performance (n = 17, 18.1%). The remaining benefits (got information regarding food assistance, class attendance increased, grades increased, able to stay enrolled) were rarely or never selected. One shopper commented "It’s a great program and it helps me get the food I need. My health and GPA have improved, and I feel less stress and have time to spend on other things. I get more sleep." This same shopper added "I am able to spend money on fruits and vegetables because the food pantry provides the staple foods like canned foods, pasta, etc." (M1) Another shopper disclosed "I ran out of money, so the pantry helped provide food for a month while there were no other options and money was low. That helped my mental and physical health by providing food when I was short on food money." (M2) Table 3 shows the frequency counts and percentages of disagree/agree responses for 13 pantry attributes.

Greater than 70% of shoppers agreed that the pantry accurately reflected three of the four attributes related to the pantry’s physical environment, i.e., organized inventory, spacious/roomy, and convenient location. However, not all shoppers were satisfied. One shopper commented "The size of pantry could be larger, and it seems disorganized." (M3) Another commented “I noticed that it was hidden and not out in the open. It’s good that it’s discrete, so that if students feel that they need to go to the food pantry, it’s not as noticeable. But, this could also be a disadvantage because it’s hidden, and some students may not know that it exists.” (F1)

The food offerings category received 55% or more agree or strongly agree ratings on three of the six attributes, i.e., food safety, familiar foods, and nutritious/healthful foods. One shopper observed "Fresh food was offered; nutritionally satisfying foods. The pantry was very clean with up-to-date food items. I didn't come across any expired or rotting food." (M3) Two shoppers expressed dissatisfaction with the variety of foods offered. "It is hard to get well-balanced meal from the pantry alone." (M1) and "There is a lot of ramen and a limited selection of foods. Cans of fruit are very common." (M2) Another shopper offered suggestions for expanding food offerings “I would like to see more of snacks, e.g., Goldfish, Cheez-Its. I’d like to see sandwiches like ham or turkey that students could take home.” (F1)

The customer service category received greater than 85% agree or strongly agree ratings on two of the three attributes, i.e., friendly service and helpful service. Shoppers commented "The environment and the people allow everyone to feel welcome." (M1), "It has a kind atmosphere." (M3), and “As far as how I was treated, I mean everybody there was very nice. I didn’t notice that any student was smothered by anyone. It is like that the students could go if they wanted to and they would be assisted if they needed any help.” (F1) One shopper expressed the concern that "The hours could be restrictive for people with a lot of class." (M1), and another suggested "It would help if there were longer hours for people with evening classes." (M2) One suggestion for improvement was "They could have a nutritional guide like MyPlate handy for guidance on how to eat a balanced meal." (M3)

## IMPLICATIONS

The findings revealed that about half the participants were food insecure, but that less than 20% used their campus pantry. This low rate of pantry use falls far below the 38% reported by El Zein et al.^21^ and the 69% reported by Rouse (see Additional Files). These differences in the rates of student pantry use may, in part, be attributable to the different data collection methods used. El Zein et al.^21^ and the present investigators administered online surveys, requiring students to add an unplanned activity to their busy schedules, which may have decreased submissions from pantry users. Rouse, in contrast, distributed the survey to shoppers at the campus food bank, making it more convenient for the students to complete the survey.

Since prolonged inadequate calorie and nutrient intakes can manifest in obesity or weight loss, decreased nutrient reserves, fatigue, cardiometabolic conditions, and mental health disorders,^4^,^7^,^17^–^19^ continued efforts from university administrators, faculty, academic advisers, and student leaders are needed to promote greater pantry use and decrease the associated stigma. Additionally, since significant negative correlations were found between the students’ AFSSM scores and their GPAs and APS scores, increasing pantry use by food insecure students could assist in protecting their health and promoting improved academic performance and retention. Accordingly, at the study site nutrition faculty include a description of the campus food pantry in course syllabi, encourage its use during the first class meeting, address the issue of stigma, and take classes to the food pantry when teaching about regional and national food insecurity and hunger in various courses. Advisers assist by identifying needy students and encouraging them to use the pantry without embarrassment, and the student dietetic and public health associations raise awareness about campus food insecurity, role model pantry use by making themselves visible at the pantry, volunteer in the operations of the pantry, and sponsor food drives.

The pantry shoppers in our study, like those surveyed by El Zein et al.^21^ and Rouse (see Additional Files), harbored primarily favorable perceptions of pantry services. To illustrate, our student shoppers observed that accessing food at the pantry allowed them to save money on their food budget which they could spend on other fixed expenses. Additionally, a strong majority of shoppers had favorable perceptions of the pantry’s physical environment and customer service. However, similar to the findings of Rouse, food offerings were regarded less favorably, suggesting less satisfaction with the variety of foods and with the availability of nutritious foods, foods for special diets, and culturally diverse foods. Since the pantry staff relies on food donations from local grocery stores and farmer’s markets and on monetary contributions to maintain its inventory, the variety and diet quality of available foods will vary from one shopping trip to the next. Furthermore, the students who were interviewed expressed several positive feelings when shopping at the pantry, including thankful, satisfied, and supported. These findings suggest that the shoppers appreciated having a campus food pantry as a resource to help them improve their food deficit and that they were treated with respect by the staff.

Food pantries can serve as temporary measures for facilitating food access only if needy students are motivated to use them. Since the present findings and work at other postsecondary institutions show low rates of pantry use,^21^ (and see Additional Files) novel approaches for lowering campus food insecurity rates are needed while continuing to operate campus food pantries. For example, the nutrition program at AppState began offering a three-credit, interdisciplinary course on skill-building for food security. Topics covered through instruction, readings, videos, discussions, and group activities include: defining food insecurity in sociocultural contexts; risk factors, health problems, and unfavorable academic impacts associated with food insecurity; coping strategies used by food insecure people; and local, state, and federal food assistance programs. The skills taught include budgeting, meal planning, food purchasing, basic cooking skills, gardening, reducing food waste, keeping food safe, and advocating for food assistance policies and programs.

Several limitations prevent the generalizability of the findings to campus pantry shoppers in Appalachia: a small sample size, data collection on a single campus, self-reporting of all measures, and the over-representation of females and white students. Additionally, the low rate of pantry use should be interpreted with caution since more students may have accessed food from the pantry but were unwilling to disclose this due to the stigma associated with pantry use reported by other investigators.^21^ The food insecurity rate of 46.2% reported for students at AppState should also be interpreted with caution given that several authors have questioned the use of the USDAERS AFSSM as an accurate measure of food insecurity among college students.^27^ These authors note that this tool has not been validated for psychometric properties in this population and that qualitative and quantitative evaluations are needed to determine the most appropriate assessment tool for obtaining an accurate prevalence of food insecurity among college students.

**SUMMARY BOX**

**What is already known about this topic?** Few studies have been published concerning college student use and perceptions of campus food pantries. Limited data suggest low pantry use due to social stigma, insufficient information about pantry policies, self-identity, and inconvenient pantry hours. Pantry characteristics that are perceived favorably by shoppers include the nutritious quality and quantity of available foods and customer service, while those perceived less favorably include the variety of food offerings, food sanitation, and pantry size.

**What is added by this report?** This study measured student use of a food pantry at a campus in Appalachia with a high rate of food insecurity, identified benefits and barriers to pantry use and pantry attributes regarded favorably by students, and offered student suggestions for pantry improvements.

**What are the implications for public health practice, policy, and research?** The low use of the campus food pantry by food insecure students suggests that these students may be jeopardizing their physical and mental health and academic success. Research is needed that investigates the roots of the stigma associated with accessing food at campus pantries. Novel policies and programs that facilitate greater access by college students to nutritious foods are needed while continuing to operate campus pantries as a short-term protective strategy.

## Supplementary Information



## Figures and Tables

**Table 1:** Reasons food insecure students have not accessed the food pantry

Reason	N	%*
Others need it more than I do	138	30.1
Feel embarrassed asking for help accessing food	95	20.7
Don’t know how to ask for help accessing food	58	12.6
I have regular adequate access to food	73	15.9
My time schedule conflicts with hours of pantry operation	45	9.8
Not interested/motivated to access food at the pantry	45	9.8
Don’t have cooking equipment	27	5.9
Won’t find any foods that I like	18	3.9
Don’t have food preparation skills	13	2.8
Other	11	2.4
Foods won't support my special diet	10	2.2
Family doesn’t want me to ask for help accessing food	9	2
Don’t have transportation to get food home	9	2
Mobility problems getting to pantry	8	1.7
Foods won’t support my religious beliefs	1	.2
Foods won’t be culturally appropriate	0	0
Don’t know how to get to the food pantry because of impaired vision	0	0

* percentages do not equal 100 because more than one option was recorded per participant

**Table 2:** Sample Demographics

Entire Sample (N = 896)		
Variable	N	%
**Food Security Status**		
Food Insecure	437	48.8
Food secure	459	51.2
**Gender**		
Male	193	21.5
Female	629	70.2
Non-cisgender	20	2.2
**Average Age**	21.1	
**Food Pantry Users (N = 94)**		
**Gender**	N	%
Male	23	27.7
Female	54	65.1
Non-cisgender	6	7.2
**Average Age**	21.8	
**Average GPA**	3.34	
**Race/ethnicity**		
African-American, not of Hispanic Origin	3	3.2
American Indian	1	1.1
Asian	1	2.6
Hispanic	7	7.4
White, Not of Hispanic Origin	60	63.8
Other	11	11.7
**Participation in SNAP/WIC**	4	4.3
**Participate in a Campus Meal Plan**	20	21.3
**Employment Status**		
Unemployed	29	30.9
One or more part-time jobs	49	52.1
One full-time job	1	1.1
Other	4	4.3
**Average Monthly Family Income**	$50,000–$74,999	
**Average Monthly Personal Income**	$0–$500	

* Not all participants responded to all questions – totals may not equal 100%

**Table 3:** Shopper perceptions of pantry attributes

Agree (strongly agree + agree)	N*	%
Foods are safe to eat	80	85.1
Familiar foods are available	78	83.0
Organized inventory	77	81.9
Friendly service	75	79.8
Helpful service	73	77.6
Spacious/Roomy	73	77.6
Convenient location	71	72.5
Open at convenient times	62	66.0
Nutritious/Healthful foods are available	58	61.7
Visually appealing	59	62.7
Variety of foods are available	55	58.5
Foods are available for special diet needs	40	42.6
Culturally diverse foods are available	26	27.7
**Disagree (strongly disagree + disagree)**	**N**	**%**
Culturally diverse foods are available	56	59.6
Foods are available for special diet needs	43	44.8
Variety of foods are available	30	32.0
Nutritious and healthful foods are available	25	26.6
Visually appealing	26	27.7
Open at convenient times	14	14.9
Convenient location	14	14.9
Spacious/Roomy	12	12.8
Helpful service	10	10.7
Friendly service	10	10.7
Organized inventory	8	8.5
Familiar foods are available	6	6.4
Foods are safe to eat	3	3.2

* percentages do not equal 100 because more than one option was recorded per participant
