# Supplementary material for: Use and Perceptions of a Campus Food Pantry Among Food Insecure College Students An Exploratory Study from Appalachia
Source: J Appalach Health. 2020 Apr 15;2(2):7–23. doi: 10.13023/jah.0202.02 (PMC9138722; doi:10.13023/jah.0202.02)

UC FOOD PANTRIES PROGRAM EVALUATION

By Rachel Rouse – UCSB

Project Goals and Basis

- **Survey current UC undergraduate and graduate students that utilized the AS Food Bank services at UCSB Goals:**
  - Evaluate the overall quality of food and services provided by UCSB's AS Food Bank
  - Identify gaps in service and areas needing improvement
  - Determine best practices for current and future food pantries
  - Identify demographics of users of UC food pantries vs. the overall student population
  - Gain insight from the students most affected by disparities in access to food

AS Food Bank Mission and Practices

- Strives to provide **friendly, confidential** service to students in need of food and toiletry items.
- Emphasizes a **holistic approach** to alleviating hunger and food insecurity through resource referral to resources that may provide more long term or comprehensive solutions
- Also offers **fresh produce, cooking demonstrations**, and occasional events outside of the pantry to distribute produce to students in Isla Vista
- Utilizes **local partnerships** with the Santa Barbara Food Bank, Isla Vista Food Co-op, and other on-campus organizations to strengthen community relationships and maximize budget
- Fundraises to fill in gaps in funding

What is Food Insecurity and How is it Measured?

- **Food insecurity** is limited or uncertain availability of nutritionally adequate and safe foods or limited or uncertain ability to acquire acceptable foods in socially acceptable ways. ~*The Journal of Nutrition*
- **According to the USDA, Food insecurity** is a socioeconomic condition, whereas hunger is a physiological state that can be caused by food insecurity. Each has a range of severities.
- **Having food security** is access by all people (in a household) at all times to enough food for an active, healthy life.

Representations of Race in this Survey vs. Student Population 2014-2015

Rust: Overall UCSB Student Population

Grey: Food Bank Survey participants

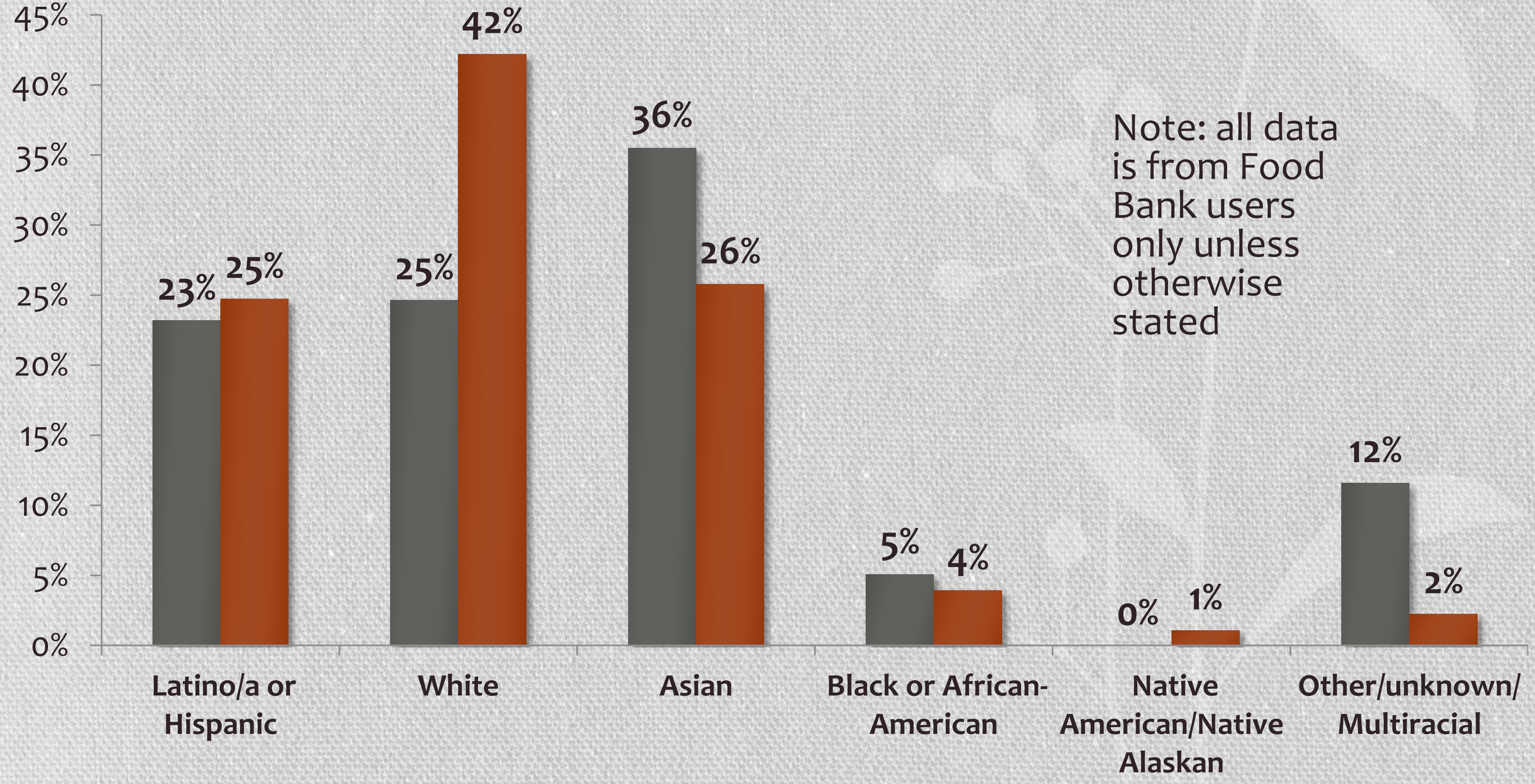

Note: all data is from Food Bank users only unless otherwise stated

Student Representation: Overall UCSB vs. Campus Food Bank

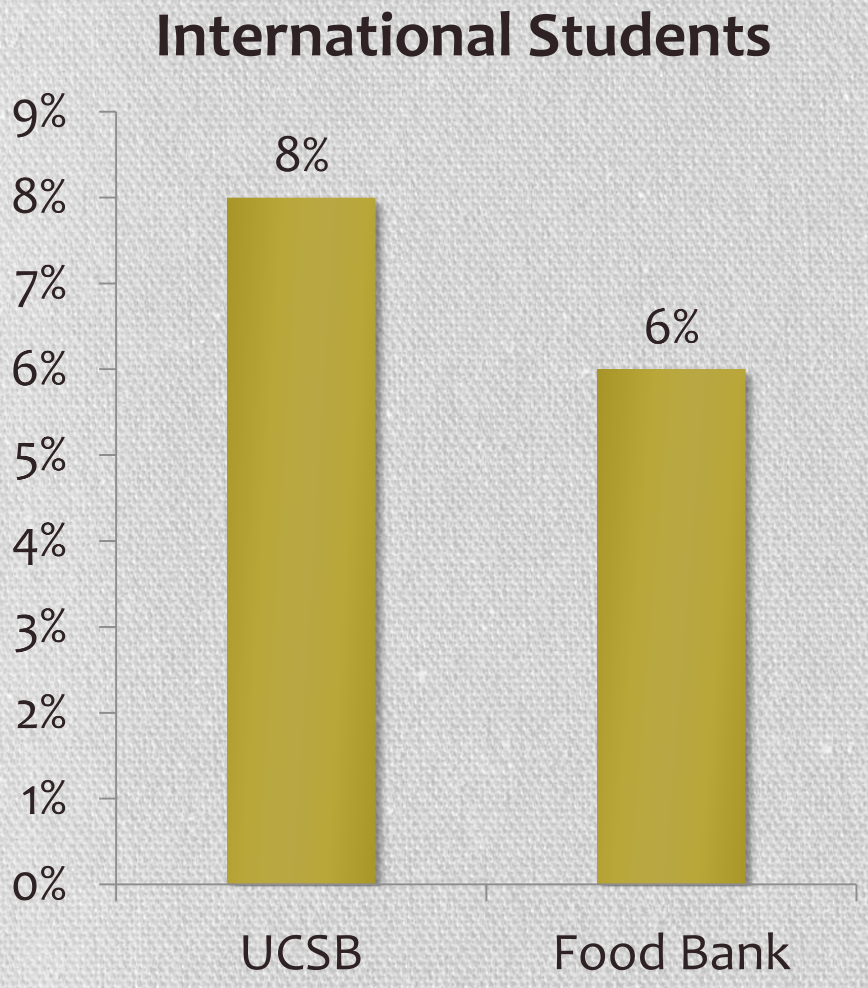

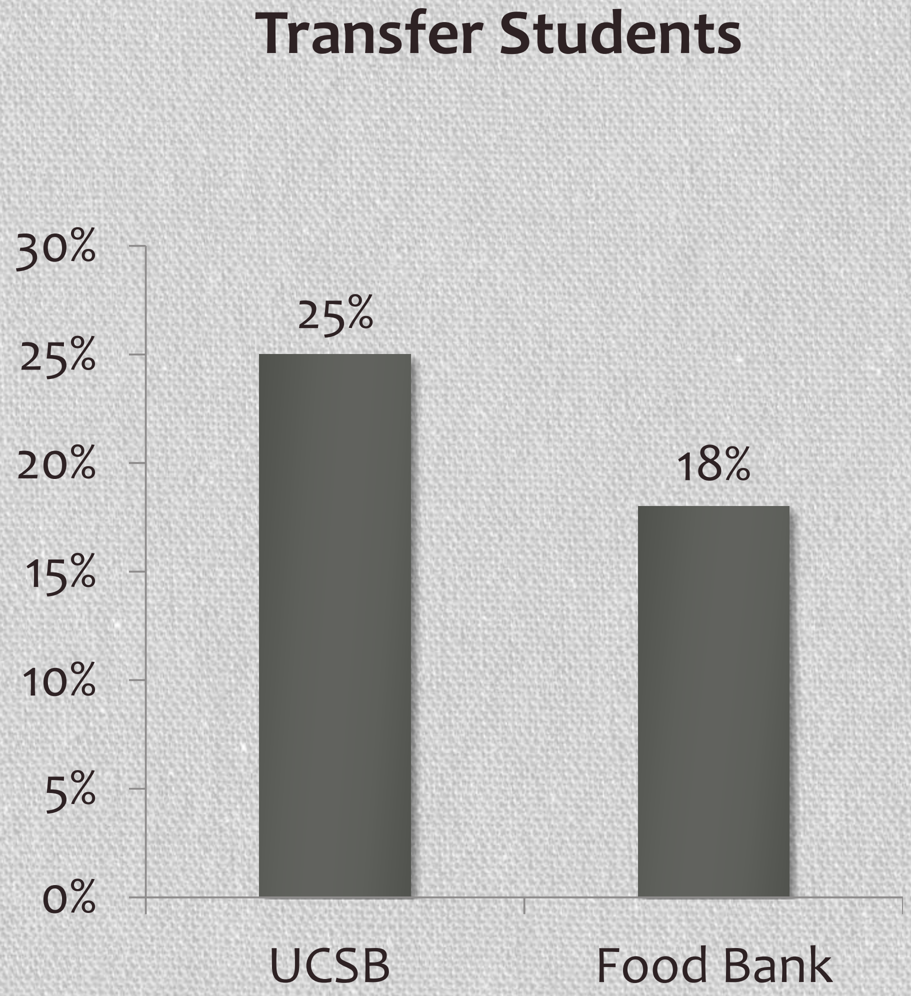

Note: all data is from Food Bank users only unless otherwise stated

Meal Skipping

43% of students using the AS Food Bank reported skipping 2 or more meals per week.

Note: all data is from Food bank users only unless otherwise stated

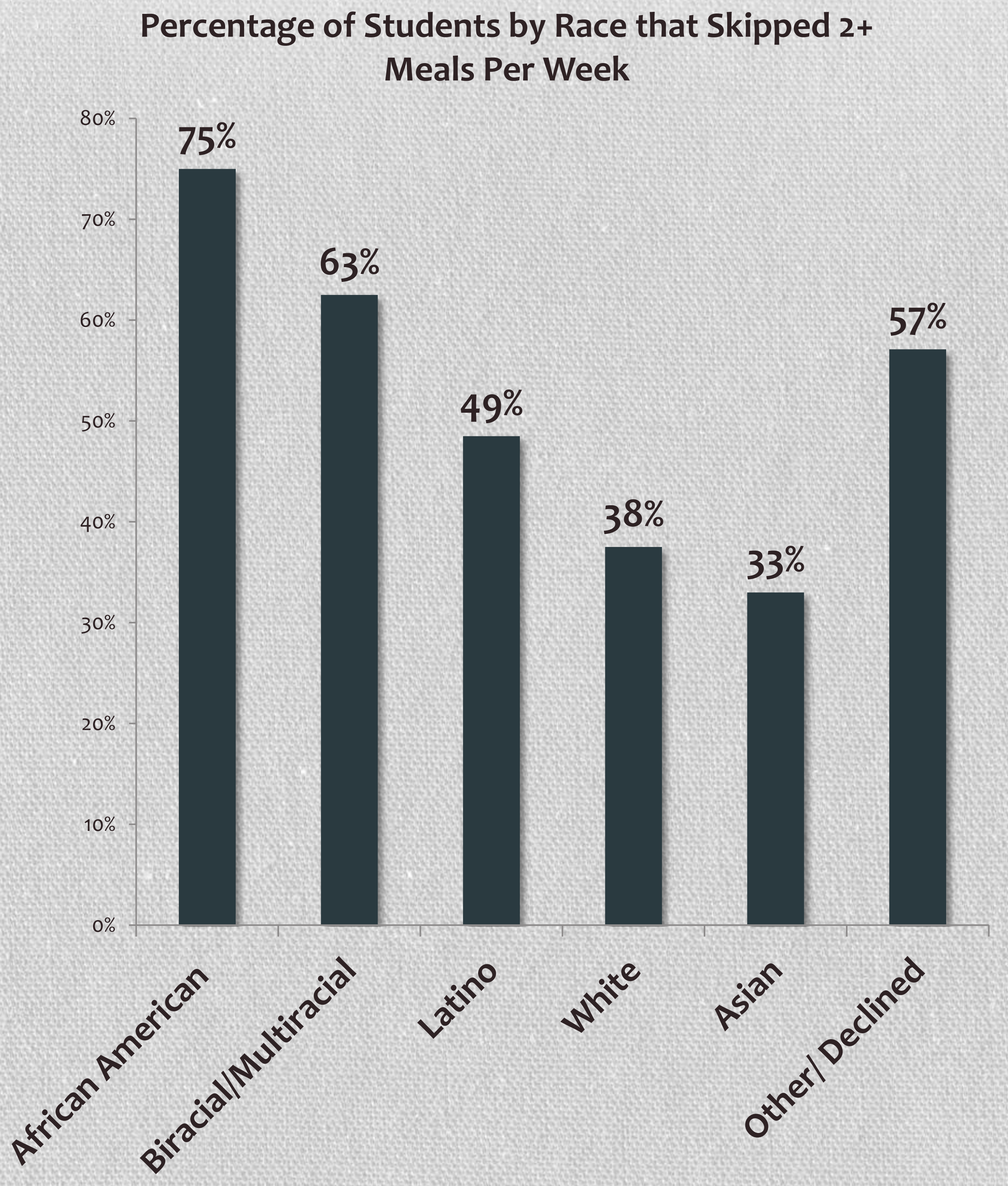

USDA Food Insecurity Module (6Q's)

Food Insecurity was determined in this project by the following USDA criteria (module):

1. The food students bought didn't last, and they didn't have money to get more.
2. Students couldn't afford to eat balanced meals.
3. Students worried whether their food would run out before they got money to buy more.
4. Students didn't eat because there wasn't enough money for food even when they were hungry.
5. Students cut the size of meals or skipped meals and did so in 3 or more months.
6. Students ate less than they felt they should.

Responses to the USDA Food Insecurity Module

- In the past 12 months **69% of AS Food Bank Users** experienced each the following conditions sometimes or often:

1. The food students bought didn't last, and they didn't have money to get more.
2. Students couldn't afford to eat balanced meals.
3. Students worried whether their food would run out before they got money to buy more.

  - **Students working 20+ hours per week are over 40% less likely to be food insecure, but the limit on working for the university is 19.5 hr/week**

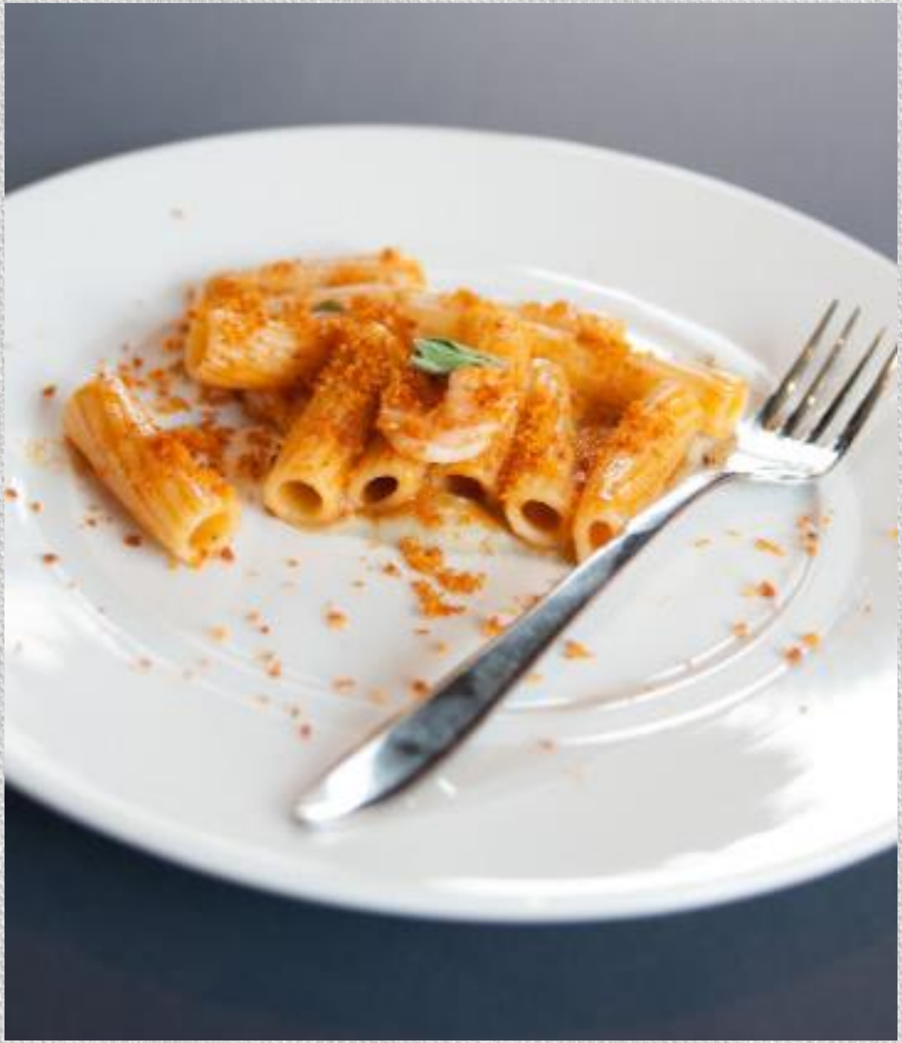

Getty Images Entertainment

USDA Module Continued:

<u>In the past 12 months:</u>

- About **44%** of students admitted that they were **hungry, but didn't eat** because there wasn't enough money for food.
- About **67% cut the size** of their meals or **skipped meals** because there wasn't enough money for food
- About **65%** of students **ate less** than they felt they should because there wasn't enough money for food
- **This is Moderately Severe Food Insecurity**

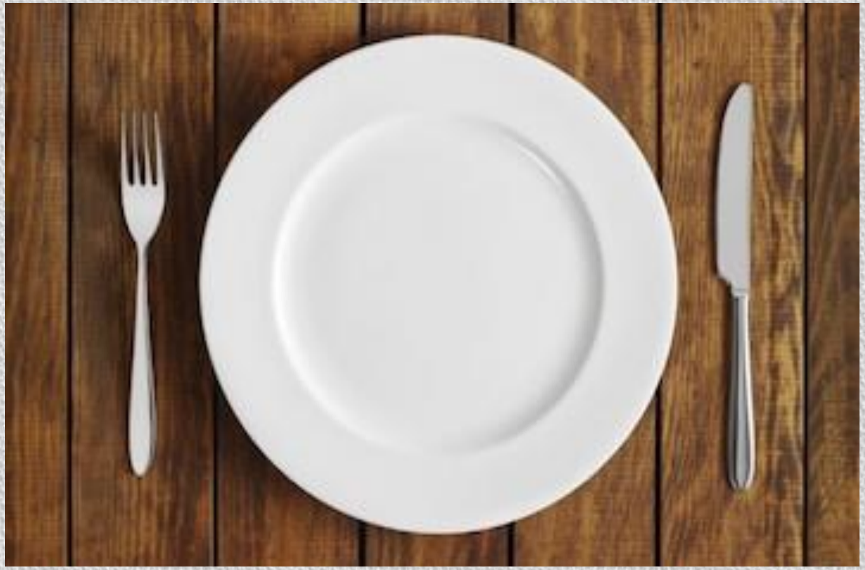

Other sources of Survey Question Development and Distribution

- **RE-AIM Model:** A successful program evaluation model; "Reach, Effectiveness, Adoption, Implementation, Maintenance"
- **UC Statewide Food Insecurity Survey:** Some questions were borrowed from this UC wide survey for comparability purposes.
- Participation in this survey was optional for students waiting to utilize the AS Food Bank services. A total of 135 surveys were collected and about 132 surveys were filled out in entirety. **All survey participants were registered undergraduate or graduate students from UCSB.**

Food Bank Usage: Frequency and Longevity

How often do you use your campus food bank/pantry? (open 3 days/week)

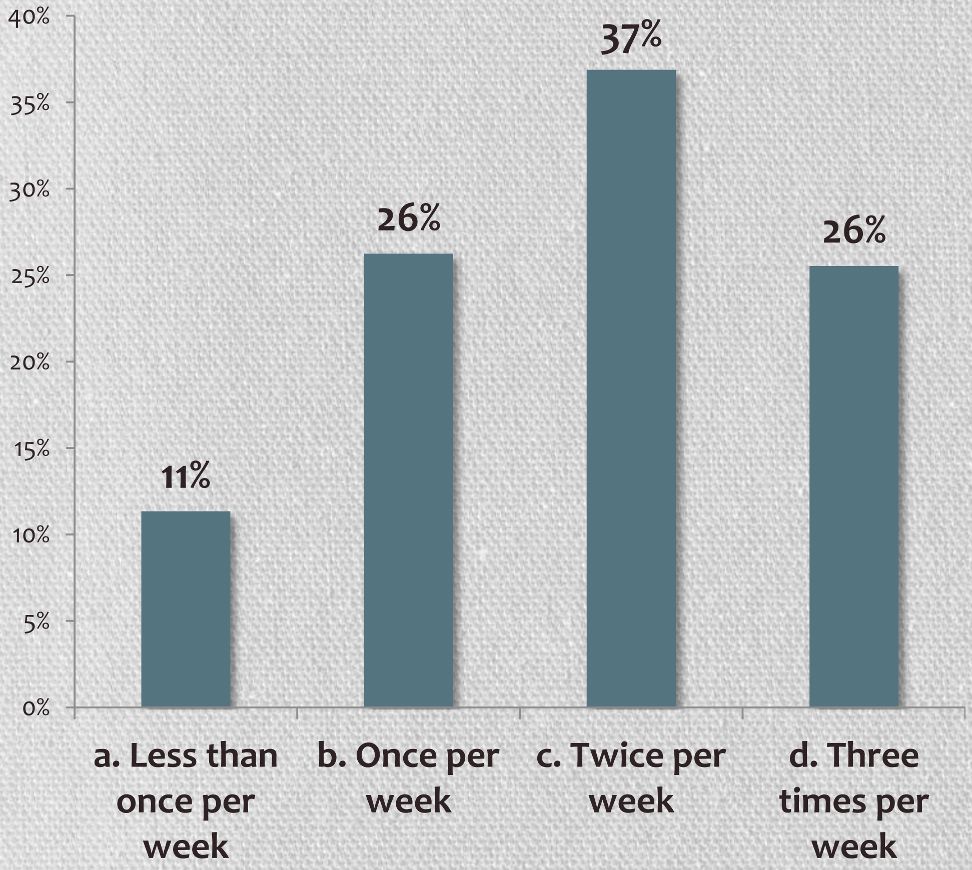

How long have you been coming to your campus food bank/pantry?

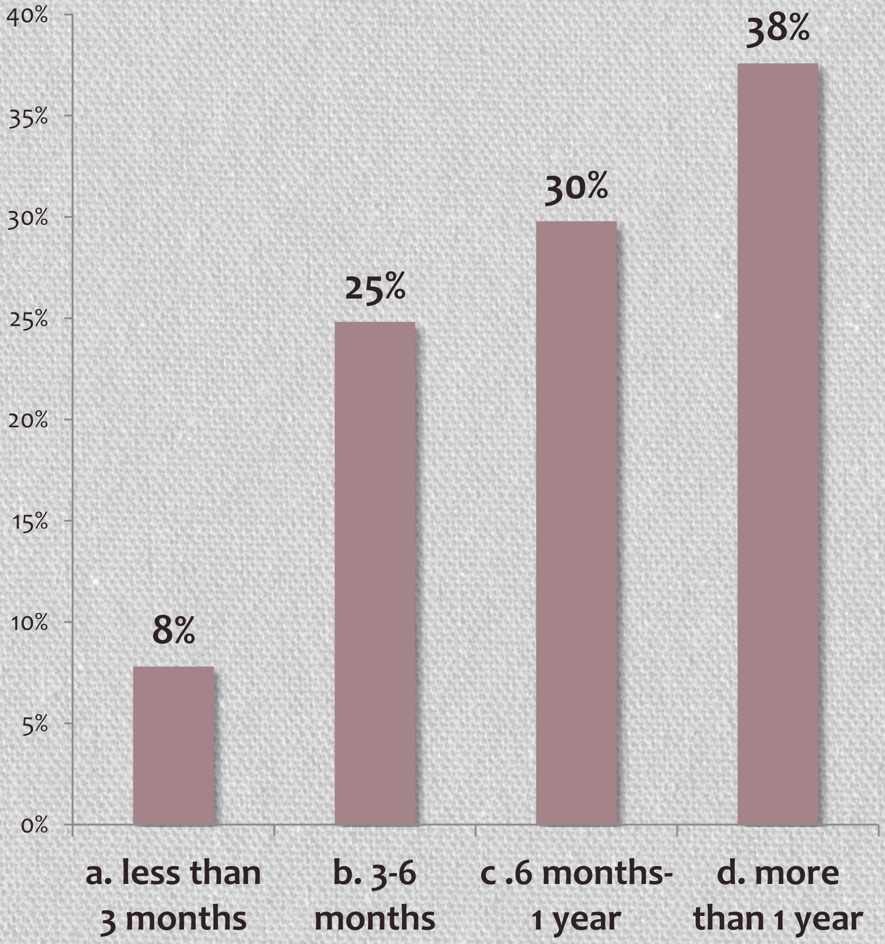

When do you find that your financial aid funds begin to run out?

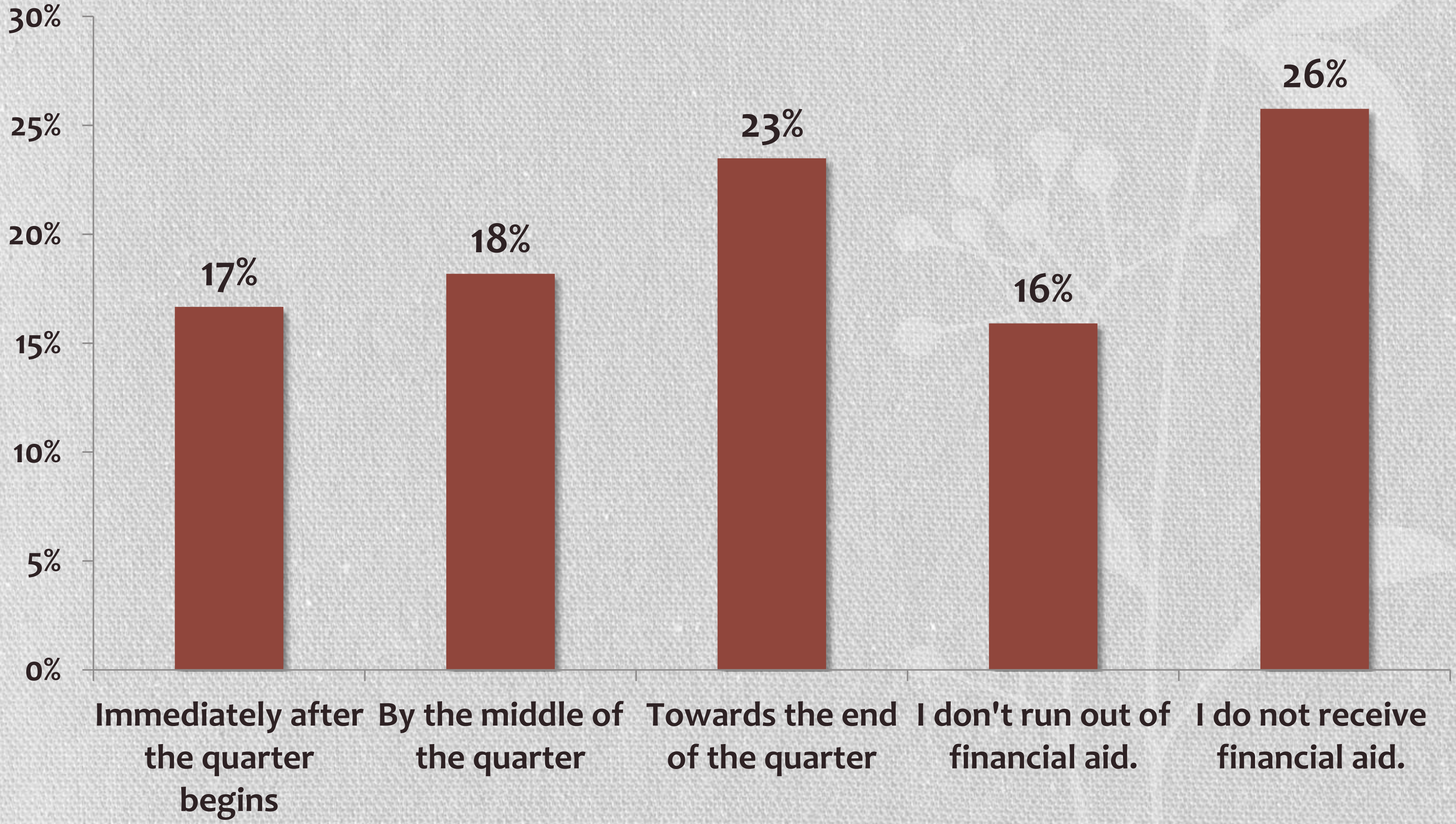

Note: all data is from Food Bank users only unless otherwise stated

Food Bank Satisfaction Ratings

- **The vast majority of students (>75%) reported high levels of satisfaction with:**
- Quality of Food Provided
- Atmosphere of the Food Bank
- Amount of Food Provided
- Quality of Services
- Wait time to get Food
- Location of the pantry
- Confidentiality of Personal Information
- Hours of Operation & Knowledgeableness of Staff

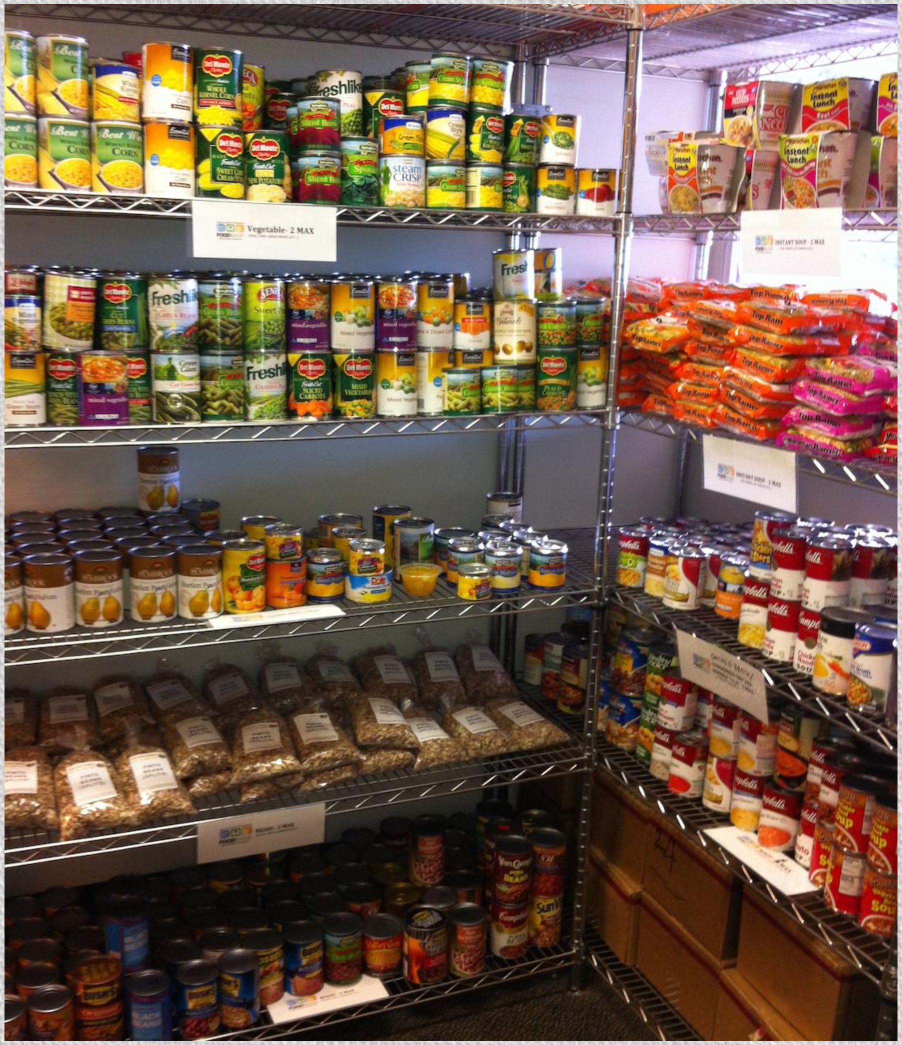

Satisfaction Ratings Continued

- **Some students reported lower satisfaction (< 60% reporting high satisfaction) with other aspects of the food bank such as:**
- Variety of Food
- Sanitation of Food
- Size of the Pantry

Many of these ratings directly result from limited funding.

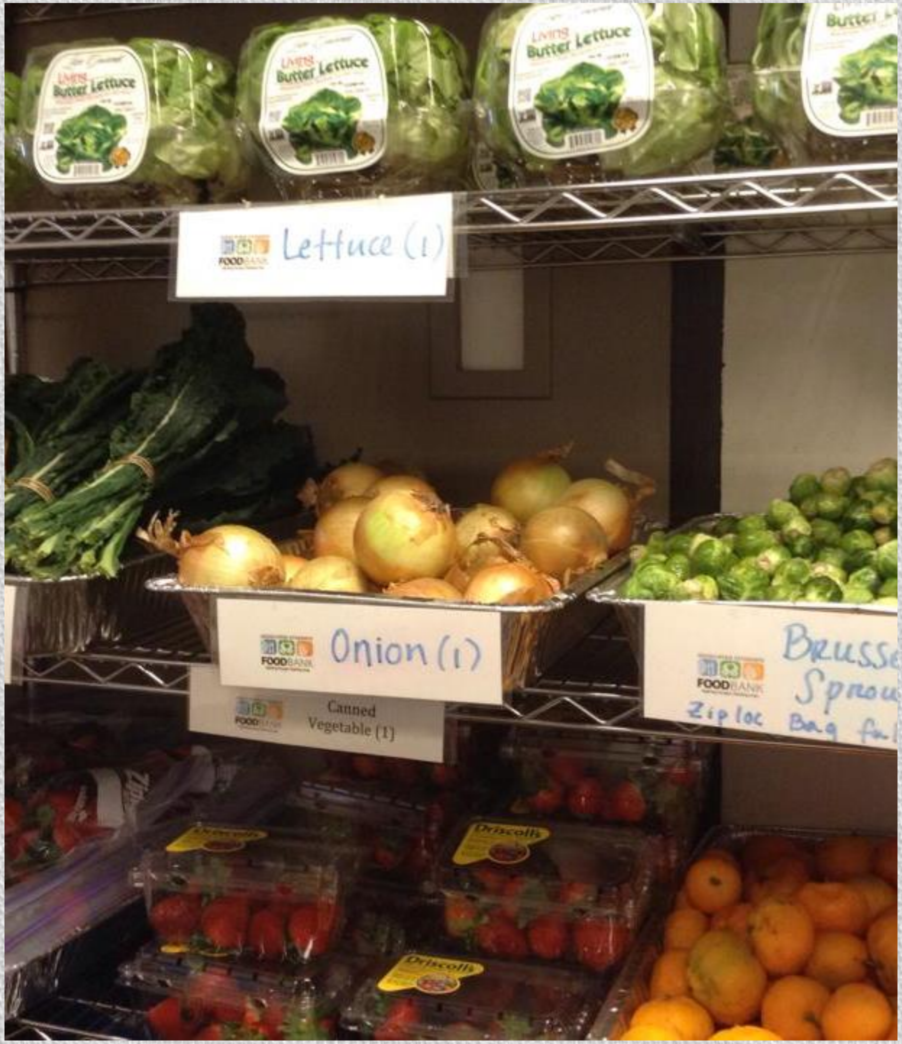

Limitations and Difficulties Encountered

UCSB's Food Bank/Pantry was the only participant in this survey because of a multitude of difficulties. These include:

- Differences in leadership- Some Pantries are run by volunteers, student government officials, or even staff members.
- Some pantries were not large enough to distribute a survey of this size
- Many pantries were difficult to reach or did not respond
- Participation from the 2 pantries that were able to distribute the survey was postponed until summer or fall
- Differences in internal pantry structure, some questions needed to be edited to fit each campus food bank/pantry

Conclusions

- The AS Food Bank is reaching the target population because **69% of users are food insecure.**
- **14.3% of U.S. Households were food insecure in 2013 (USDA)**
- Data shows that students are highly dependent on this service for their longer terms needs
- The AS Food Bank is only a temporary fix for a more systematic issue
- More work is needed to find longer term solutions such as Cooking Workshops and Calfresh referral, increasing food literacy may maximize financial aid $$

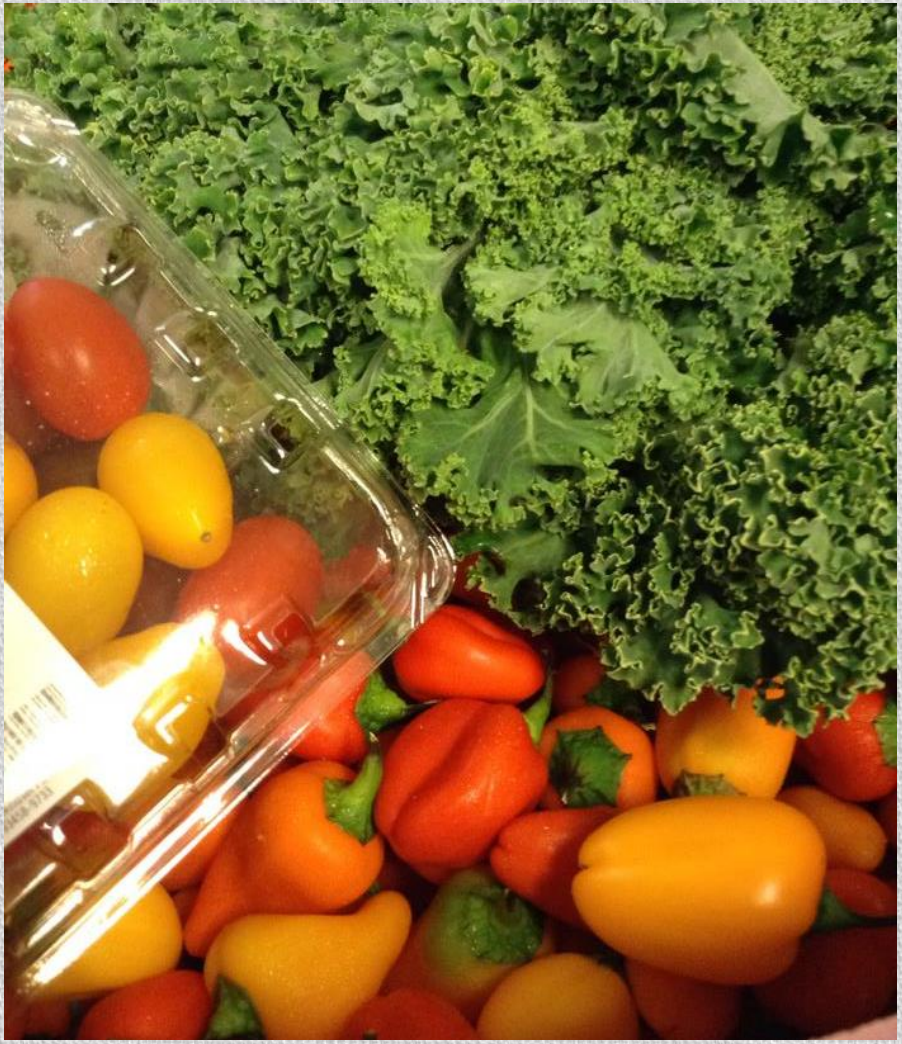

Supplement: Supplementary file 1 [file 1038UCFoodPantriesMacArthur2.2.2.pdf]
